# A ubiquitin fusion reporter to monitor muscle proteostasis in *C. elegans*

**DOI:** 10.17912/micropub.biology.000824

**Published:** 2023-04-21

**Authors:** Carl Elias Kutzner, Karen Carolyn Bauer, Thorsten Hoppe

**Affiliations:** 1 Institute for Genetics, University of Cologne, Cologne, Germany; 2 Cologne Excellence Cluster on Cellular Stress Responses in Aging Associated Diseases (CECAD), Cologne, Germany; 3 Center for Molecular Medicine (CMMC), Cologne, Germany

## Abstract

Muscle is a highly dynamic tissue in which a variety of folding and degradation processes are active to maintain protein homeostasis (proteostasis) and functionality. The muscle-specific chaperone UNC-45 folds the motor protein myosin and assembles it into myofilaments. Malfunction of this chaperone leads to misfolding of myosin, disorganization of myofilaments, and degradation of misfolded myosin molecules by the proteasome. Here, we present a new muscle-specific ubiquitin fusion degradation (UFD) model substrate in *C. elegans* that helps clarify how UNC-45 dysfunction affects muscle proteostasis.

**Figure 1. A muscle-specific ubiquitin fusion degradation (UFD) model substrate for monitoring myosin-associated proteostasis:**
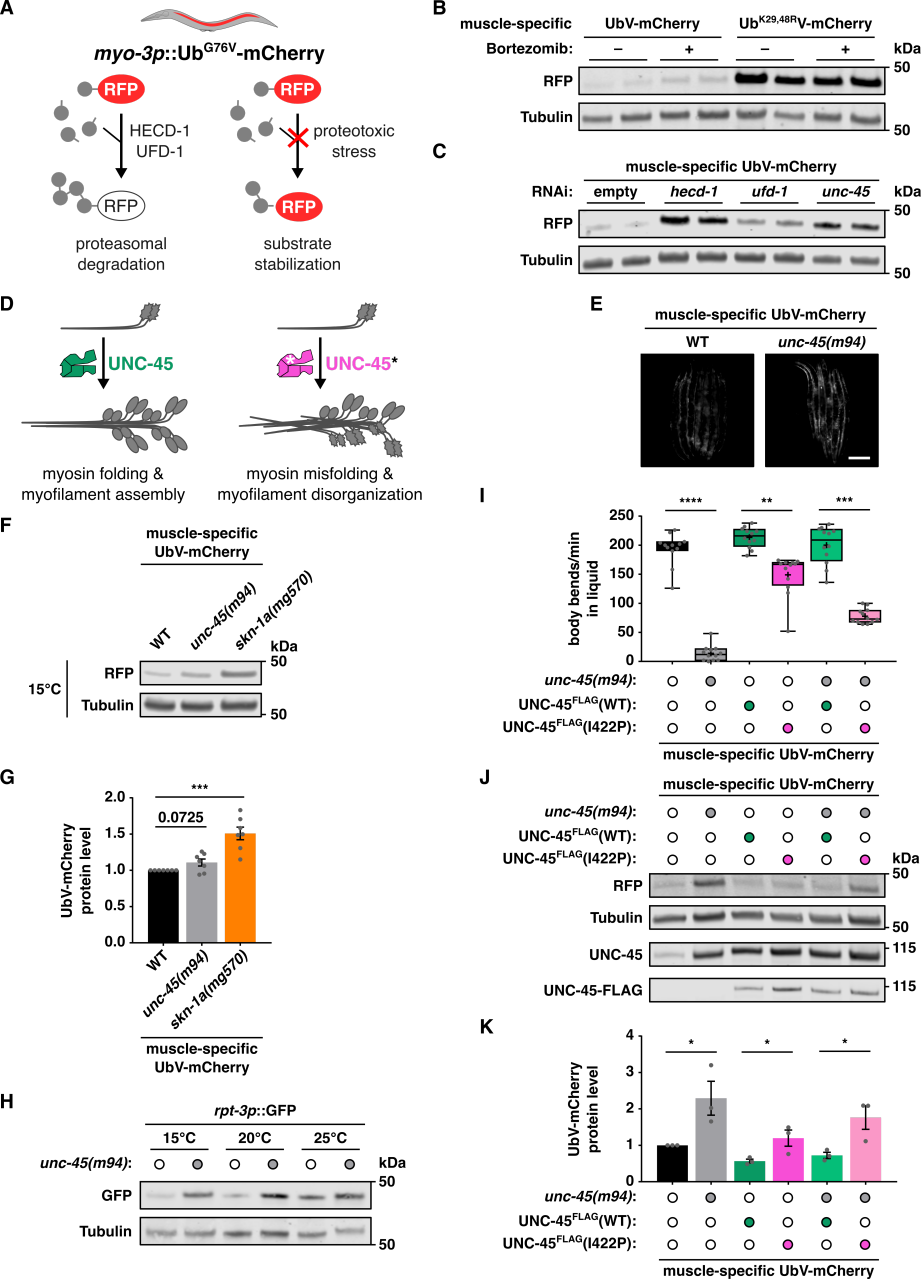
( **A** ) The UFD model substrate UbV-mCherry monitors ubiquitin-dependent proteasomal degradation specifically in the *C. elegans* body wall muscle (BMW). ( **B** , **C** ) The muscle-specific UFD model substrate is stabilized by proteasome inhibition using bortezomib ( **B** ) and RNAi depletion of indicated regulators ( **C** ). Representative Western blots of duplicate (n = 2) worm lysates with the indicated treatments for detection of UbV-mCherry (RFP) and tubulin. ( **D** ) The myosin chaperone UNC-45 folds myosin and assembles it into myofilaments. Loss-of-function (lof) mutations in UNC-45 (*) lead to misfolding and disorganization of myosin. ( **E** ) The muscle-specific UFD model substrate is stabilized by a temperature-sensitive (ts) *unc-45(m94)* allele that leads to myosin misfolding and increased proteasomal degradation at elevated cultivation temperatures. WT: wild-type. Scale bar: 200 μm. ( **F** ) At the permissive temperature, the muscle-specific UFD model substrate is only slightly stabilized by the *unc-45(m94)* ts allele, but more strongly stabilized by a *skn-1a(mg570)* lof allele. Representative Western blot of worm lysates with the indicated genotypes for detection of UbV-mCherry (RFP) and tubulin. ( **G** ) Quantification of UbV-mCherry bands in Western blots from n = 7 independent experiments. Data show means ± SEM of n = 7 independent experiments; ***p < 0.001; ratio paired T-test. ( **H** ) A transcriptional reporter under control of the SKN-1A-responsive proteasomal subunit gene promoter *rpt-3* is upregulated by the *unc-45(m94)* allele at all temperatures. Representative Western blot of worm lysates with the indicated genotypes and treatments detecting GFP and tubulin. ( **I** ) Transgenic expression of the human pathogenic variant *Ce* UNC-45(I422P) corresponding to *Hs* UNC-45B(S403P) shows a dominant negative effect on motility of adult worms. Data show mean values ± SEM obtained from n = 3 independent experiments; **p < 0.01, ***p < 0.001, ****p < 0.0001; one-way ANOVA with Dunn’s post-hoc test. ( **J** , **K** ) Transgenic expression of the human pathogenic variant *Ce* UNC-45(I422P) stabilizes the muscle-specific UFD model substrate already in the WT genetic background. ( **J** ) Representative Western blot of worm lysates with indicated genotypes detecting the muscle-specific UFD substrate (RFP), tubulin, total UNC-45 and transgenic UNC-45-FLAG. ( **K** ) Quantification of UbV-mCherry signals in Western blots of n = 3 biological replicates. Data show mean values ± SEM obtained from n = 3 independent experiments; *p < 0.05; ratio paired T-test.

## Description

Muscle is a highly dynamic, energy-consuming, and metabolically active tissue in which a plethora of folding and degradation pathways are active to maintain protein homeostasis (proteostasis) and functionality (Höhfeld et al. 2021). Protein folding is mediated by molecular chaperones such as UNC-45, the myosin-specific chaperone in muscle (Barral et al. 1998, Ao and Pilgrim 2000, Gazda et al. 2013, Lee et al. 2014) . Dysfunction of UNC-45 leads to myosin misfolding, myofilament disorganization, and increased proteasomal degradation of misfolded myosin molecules, which impairs muscle function (Epstein and Thomson 1974, Barral et al. 2002, Landsverk et al. 2007, Gaiser et al. 2011, Gazda et al. 2013, Bujalowski et al. 2015, Hellerschmied et al. 2019) . In this context, we recently described pathogenic variants in the *UNC45B* gene that cause progressive myopathy in humans (Donkervoort et al. 2020) . Degradation of misfolded or superfluous proteins is performed by the ubiquitin-proteasome system (UPS, Liebl and Hoppe 2016). In the ubiquitin fusion degradation (UFD) pathway, a cascade of ubiquitin-activation (E1), ubiquitin-conjugation (E2) and ubiquitin-ligase (E3) enzymes attach ubiquitin to target proteins and mark them for degradation (Kwon and Ciechanover 2017) . To date, various degradation co-factors and conditions have been found with UFD model substrates in cell culture and *in vivo* , e.g. in *C. elegans* (Menéndez-Benito et al. 2005, Liu et al. 2011, Segref et al. 2011, Segref et al. 2014, Matilainen et al. 2016, Finger et al. 2018, Lehrbach and Ruvkun 2019, Ravanelli et al. 2022, Segref et al. 2022). However, muscle-specific analyses of proteostasis using *in vivo* UFD model substrates are still lacking. Here, we present a tissue-specific UFD model substrate in *C. elegans* body wall muscle (BWM). We use this model substrate to elucidate myosin proteostasis in the muscle supported by UNC-45.

We express the fluorophore mCherry, N-terminally tagged with a noncleavable ubiquitin (Ub(G76V)), under the BWM-specific *myo-3* promoter (UbV-mCherry, Figure 1A ). Under non-stress conditions, poly-ubiquitylation of the ubiquitin moiety triggers degradation of the UFD model substrate by the 26S proteasome (Dantuma et al. 2000, Segref et al. 2011) . The substrate can be stabilized at the protein level when lysine residues 29 and 48 in ubiquitin are mutated to arginine, preventing further ubiquitylation (Ub ^K29,48R^ V-mCherry, Figure 1B ). To test the functionality of the new substrate, we blocked its degradation by treatment with the proteasome inhibitor bortezomib or by RNAi-mediated depletion of UFD regulators (the E3 ligase HECD-1 and the p97/CDC-48-associated co-factor UFD-1). We observed stabilization of the UbV-mCherry protein, although not to the same extent as the substrate lacking the lysine residues 29 and 48 ( Figure 1B and 1C), suggesting redundancies in proteostasis pathways in muscle, such as the heat shock response or autophagy (Ben-Zvi et al. 2009, Suraweera et al. 2012, van Oosten-Hawle et al. 2013, Lehrbach and Ruvkun 2019, Alexander-Floyd et al. 2020) . To test a muscle-specific proteostasis factor, we turned to the Hsp90 co-chaperone UNC-45, which folds and assembles translated myosin proteins in the growing thick filament (Gazda et al. 2013, Lee et al. 2014) . In higher organisms and *in vitro* , UNC-45 has been observed to help refold and stabilize misfolded myosin molecules (Etard et al. 2008, Bujalowski et al. 2015) . Polymorphisms in *unc-45* or the myosin gene *unc-54* , which produce temperature-sensitive (ts) proteins in *C. elegans* , lead to myosin misfolding and disorganization ( Figure 1D, Epstein and Thomson 1974, Macleod et al. 1977, Barral et al. 1998). We induced UNC-45 dysfunction by RNAi-mediated depletion of *unc-45* or by the *unc-45(m94)* ts mutant and observed stabilization of the UbV-mCherry protein specifically in BWM ( Figure 1C and 1E). Since myosin is the major muscle protein, impairment of its folding by UNC-45 may lead to UPS overload in muscle.

Misfolded myosin species, but not complete knockout, activate the transcription factor SKN-1A, which in turn adjusts proteasomal capacity to the increased demand for degradation by inducing expression of proteasomal subunit genes, e.g., *rpt-3* (Lehrbach and Ruvkun 2019) . To confirm the same pathway for myosin misfolding triggered by UNC-45 dysfunction, we used a reporter strain for proteasomal subunit expression (Lehrbach and Ruvkun 2016) . We observed that the *unc-45(m94)* ts allele activates expression of the SKN-1A activity reporter *rpt-3p* ::GFP already at the permissive temperature ( Figure 1H ), when the muscle-specific UFD model substrate is only slightly stabilized ( Figure 1F and 1G). When grown at the permissive temperature of 15°C, *unc-45(m94)* are superficially wild-type. Our results indicate that in a background of UNC-45 dysfunction, proteasomal capacity is adjusted to compensate for impairments in the proteostasis network in muscle.

Finally, to use the novel muscle-specific UFD model substrate in *C. elegans* to study human disease pathology, we analyzed an available transgenic strain of a human pathogenic variant that exhibits a dominant negative phenotype. Transgenic expression of the *C. elegans* ortholog *Ce* UNC-45(I422P) of the human pathogenic variant *Hs* UNC-45B(S403P) reduces wild-type worm motility and stabilizes the muscle-specific UFD model substrate in the wild-type and *unc-45(m94)* background compared with an UNC-45(WT) transgene. Overall, UNC-45 dysfunction impairs the proteostasis network in muscle via SKN-1A and the proteasome. Dysregulation of proteasomal degradation of muscle proteins is a possible mechanism for how human pathogenic variants of *UNC45B* lead to a progressive myopathy phenotype.

Further studies will clarify which other proteostasis factors are involved in the response to myosin misfolding in muscle in addition to the chaperone UNC-45, the proteasome, and the transcription factor SKN-1A. The muscle-specific UFD model substrate and other newly developed techniques will allow us to determine how misfolded myosin affects the proteostasis network in muscle and which E3 ligases and accessory factors are involved in myosin degradation in the *C. elegans* BWM.

## Methods

*C. elegans* maintenance

Nematodes were maintained at 15°C on plates containing nematode growth medium (NGM) seeded with *E. coli* OP50 as food source and grown at 20°C (unless otherwise indicated) for experiments using standard procedures and methods (Brenner 1974, Stiernagle 2006) . The N2 Bristol strain (Caenorhabditis Genetics Center) served as wild-type (WT) background.

RNA interference (RNAi)

RNAi using the feeding method established for *C. elegans* was performed as described (Kamath et al. 2001) . Age-synchronized worms were transferred to RNAi plates seeded with *E. coli* HT115 bacteria expressing the respective double-stranded RNA (dsRNA). The RNAi clones were from the RNAi Collection (Ahringer). As a control, bacteria transformed with the empty pPD129.36 vector were used for feeding.

Bortezomib treatment

Age-synchronized worms were grown to L3 stage at 20°C and transferred to plates containing 10 µM bortezomib (Selleckchem) seeded with *E. coli* OP50 bacteria. As controls, plates containing an equal volume of the solvent DMSO were used.

Microscopy

For microscopy, 5-10 individual adult worms grown at 20°C were paralyzed in levamisole on 2 % agar pads on slides and imaged using an AXIO Zoom.V16 microscope (Zeiss).

Western blotting

For whole worm lysates, ~250 animals were collected in 30 μl 1x SDS loading buffer. Samples were then boiled at 95°C for 5 min, sonicated (30 s at 60 % amplitude), and boiled again at 95°C for 5 min. Samples were then centrifuged at 20,000 g for 1 min. For Western blotting, equal amounts of protein were separated by SDS-PAGE using self-poured Bis-Tris acrylamide gels in MES running buffer. Protein transfer was performed in a semi-dry blotting system (Bio-Rad, Trans-Blot Turbo) with NuPAGE® transfer buffer. Antibodies were diluted in 1x Roti®-block (Carl Roth). Visualization of fluorescent signals was performed using Odyssey scanner (LI-COR) and the Image Studio Lite v5.2 software. All Western blots were repeated in at least n = 3 independent experiments.

Motility assay

For body bend assays, individual young adult worms grown at 20°C were placed in 1 ml M9 buffer (room temperature 21°C), and body bends were counted for 30 s and doubled to calculate the number of body bends per minute.

## Reagents

List of *C. elegans* strains

**Table:** 

**Strain**	**Referred to as**	**Genotype**	**Available from**
PP3159	WT; UbV-mCherry	*hhIs232[Pmyo-3::UbV-mCherry, unc-119(+)]*	Hoppe lab
PP3021	Ub(K29,48R)V-mCherry	*unc-119(ed4)III; hhIs230[Pmyo-3::K29,48R-UbV-mCherry, unc-119(+)]*	Hoppe lab
PP3366	*unc-45(m94)* ; UbV-mCherry	*unc-45(m94)III; hhIs232[Pmyo-3::UbV-mCherry, unc-119(+)]*	Hoppe lab
PP3460	*skn-1a(mg570)* ; UbV-mCherry	*skn-1a(mg570)IV; hhIs232[Pmyo-3::UbV-mCherry, unc-119(+)]*	Hoppe lab
GR2183	*rpt-3p* ::GFP	*mgIs72[rpt-3p::GFP + dpy-5(+)]II*	CGC Lehrbach and Ruvkun 2016
PP3572	*unc-45(m94)* ; *rpt-3p* ::GFP	*unc-45(m94)III; mgIs72[rpt-3p::GFP + dpy-5(+)]II*	Hoppe lab
PP3408	UNC-45-FLAG(WT); UbV-mCherry	*hhIs84[unc-119(+); unc-54::unc-45::FLAG]; hhIs232[Pmyo-3::UbV-mCherry, unc-119(+)]*	Hoppe lab
PP3367	UNC-45-FLAG(I422P); UbV-mCherry	*hhIs254[Punc-54::unc-45(I422P)::FLAG; unc-119(+)]; hhIs232[Pmyo-3::UbV-mCherry, unc-119(+)]*	Hoppe lab
PP3409	*unc-45(m94)* ; UNC-45-FLAG(WT); UbV-mCherry	*unc-45(m94)III; hhIs84[unc-119(+); unc-54::unc-45::FLAG]; hhIs232[Pmyo-3::UbV-mCherry, unc-119(+)]*	Hoppe lab
PP3365	*unc-45(m94)* ; UNC-45-FLAG(I422P); UbV-mCherry	*unc-45(m94)III; hhIs254[Punc-54::unc-45(I422P)::FLAG; unc-119(+)]; hhIs232[Pmyo-3::UbV-mCherry, unc-119(+)]*	Hoppe lab

List of antibodies and chemicals:

**Table:** 

**Antibody**	**Source**	**Identifier**
Mouse monoclonal antibody (6G6) to Red Fluorescent Proteins	ChromoTek	Cat# 6g6 RRID: AB_2631395
Mouse monoclonal anti-alpha Tubulin (Clone B-5-1-2)	Sigma-Aldrich	Cat# T6074 RRID: AB_477582
Living Colors® A.v. monoclonal antibody (JL-8) (Mouse anti-GFP)	Clontech	Cat# 632380 RRID: AB_10013427
Purified rabbit anti-UNC-45 (rabbit #9624)	Biogenes	Not commercially available, Hoppe Lab
Monoclonal anti-FLAG M2 antibody produced in mouse	Sigma-Aldrich	Cat# F3165 RRID: AB_259529
Donkey anti-mouse IRDye® 800CW	LI-COR	Cat# 926-32212 RRID: AB_621847
Donkey anti-rabbit IRDye® 680CW	LI-COR	Cat# 926-32223 RRID: AB_621845
**Chemical**	**Source**	**Identifier**
Bortezomib (PS-341)	Selleckchem	Cat# S1013
